# Decoding of Visual Attention from LFP Signals of Macaque MT

**DOI:** 10.1371/journal.pone.0100381

**Published:** 2014-06-30

**Authors:** Moein Esghaei, Mohammad Reza Daliri

**Affiliations:** 1 School of Cognitive Sciences (SCS), Institute for Research in Fundamental Sciences (IPM), Tehran, Iran; 2 Biomedical Engineering Department, Faculty of Electrical Engineering, Iran University of Science and Technology (IUST), Tehran, Iran; 3 Cognitive Neuroscience Laboratory, German Primate Center (DPZ), Goettingen, Germany; McGill University, Canada

## Abstract

The local field potential (LFP) has recently been widely used in brain computer interfaces (BCI). Here we used power of LFP recorded from area MT of a macaque monkey to decode where the animal covertly attended. Support vector machines (SVM) were used to learn the pattern of power at different frequencies for attention to two possible positions. We found that LFP power at both low (<9 Hz) and high (31–120 Hz) frequencies contains sufficient information to decode the focus of attention. Highest decoding performance was found for gamma frequencies (31–120 Hz) and reached 82%. In contrast low frequencies (<9 Hz) could help the classifier reach a higher decoding performance with a smaller amount of training data. Consequently, we suggest that low frequency LFP can provide fast but coarse information regarding the focus of attention, while higher frequencies of the LFP deliver more accurate but less timely information about the focus of attention.

## Introduction

Attention as a filtering mechanism selects behaviorally relevant stimuli for more effective processing in the mammalian cortex. The neural correlates of this mechanism, especially in the visual system, have been intensively studied during the last few decades both in humans and non-human primates [1]–[3]. The most basic neuronal correlate of attention has been reported as the increase in firing rate of neurons that are selective to the feature or position that the animal has attended to. This effect has been reported in different visual areas of macaques [3], [4]. Specifically, when attention is directed toward a stimulus located inside the receptive field (RF) of a neuron, the firing rate of that neuron increases relative to when attention is toward a similar stimulus outside the RF.

The local field potential (LFP) is another signature of neural activity which has recently been paid considerable attention in neuroscience. LFP mainly represents synaptic activities of cortical neurons, as well as other factors such as calcium spikes and membrane oscillations [5]. Different frequencies of LFPs have been studied under various cognitive states and the results show that high frequencies (>30 Hz) mostly represent local neural activities indicating stimulus processing, while lower frequencies (<20 Hz) show wide-range activities shared between large populations of neurons [6]. Attention has also been shown to influence LFP signals in different sensory areas of the monkey cortex [7]–[13]. Previous studies have shown that spatial attention is correlated with an increase in high frequency oscillations and decrease in low frequency oscillations in LFPs of the visual cortex [8]–[10], albeit see [13] for different results. Low frequency and high frequency oscillations are linked to each other through a mechanism known as phase-amplitude coupling [14]–[16]; the phase of low frequency oscillations (<20 Hz) determines the high frequency power (30–200 Hz) across a variety of cortical areas in different species [17]. It is assumed that the brain exploits this mechanism to control local computations in cortical areas [15], [17]. Attention also influences the phase of on-going low frequency oscillations of the LFP in monkey visual cortex [11]. Therefore, it is assumed that attention modulates local cortical activities by controlling the phase of low frequency oscillations which are functionally involved in the processing of stimuli [15], [17].

The influence of attention on the power of LFP signals in different frequencies would suggest it to be a helpful signal for decoding attentional state. This can be used in BCIs, devices that use brain signals to decode the intention of human and non-human primates. These devices can be incorporated into prostheses such as artificial limbs [18]. BCIs may use invasive or non-invasive methods for decoding. Non-invasive techniques compromise EEG, fMRI, MEG and near-infrared spectroscopy while invasive methods use single cell spikes and LFPs.

Non-invasive BCIs can be used for healthy humans since they do not involve any surgical procedures. In contrast to fMRI and MEG, EEG and near-infrared spectroscopy are more relevant options to be used for every-day applications. Specifically, EEG signals have been extensively investigated to control matrix spellers [19], [20]; for instance, analysis of modulations of the P300 has led to high performance in decoding the letter on the screen to which the subject has attended. However, the main shortcoming of this technique is the long time needed to decode each character due to multiple presentations of the matrix which are needed to evoke reliable signals. Invasive methods on the other hand can perform the decoding at a higher speed since there is much more spatial resolution and also higher signal-to-noise ratio [21].

Invasive methods have been used to decode brain signals in both rodents and primates. There have been numerous studies showing that movement direction in non-human primates can be decoded using intra-cortical signals [22]–[24], especially LFP recorded from the motor cortex [25], [26]. Slutzky et al. [27] showed that the forelimb movement of rats could be decoded using LFP recorded from their sensorimotor cortex. LFPs recorded from the visual cortex (inferior temporal cortex) of macaque monkeys has also been used to decode visual stimuli presented to them [28], [29]. Similarly, Manyakov et al. [30] decoded stimulus-reward pairing using LFPs recorded from macaque V4. Smith et al. [31] also decoded vocalizations from LFPs recorded from auditory areas of the monkey cortex. However, it has not yet been explored how invasive recordings could be used for decoding the allocation of visual attention. Nevertheless, Rotermund et al. [32] have used the semi-invasive technique of the electro-corticogram (ECoG) to decode the focus of attention in macaques and have shown that gamma frequency can decode attention at high performance, while low frequencies cannot decode attention better than chance [32].

We investigated whether LFP recorded from the visual cortex (medial temporal area MT) of a macaque monkey can be used to decode the focus of attention, and compared the contribution of different frequency bands to the performance of decoding. We found that the power of LFPs at different frequencies can be used to decode the focus of attention with appreciable performance, where gamma frequencies (31–120 Hz) had the highest performance.

## Materials and Methods

### Ethics Statement

All procedures of this study have been approved by the regional government office (Niedersächsisches Landesamt für Verbraucherschutz und Lebensmittelsicherheit (LAVES)). The animal was group-housed with other macaque monkeys in facilities of the German Primate Center in Goettingen, Germany in accordance with all applicable German and European regulations. The facility provides the animals with an enriched structured environment (incl. toys and wooden structures), exceeding the size requirements of the European regulations, including access to outdoor space.

The craniotomy and head post implantation were done under full anesthesia and with appropriate analgesics. The German Primate Center has several veterinarians on staff that monitor and examine the animals and consult on any procedures.

During the study the animal had unrestricted access to fluid, except on the days where data were collected or the animal was trained on the behavioral paradigm. On these days the animal was allowed unlimited access to fluid through his performance in the behavioral paradigm. Here he received fluid rewards for every correctly performed trial. Throughout the study the animal's psychological and medical welfare was monitored by the veterinarians, the animal facility staff and the lab's scientists, all specialized on working with non-human primates.

### Behavioral Task and Recording

**Figure 1:**
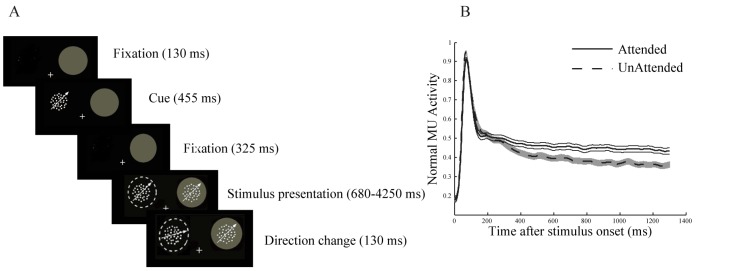
Behavioral paradigm and attentional modulation of MUA. A. A trial started when the monkey touched a lever and fixated the central fixation point. The trial would abort without any reward if the monkey released the lever or broke its fixation at any time. After a short (130 ms) blank screen a static RDP was shown for a 455 ms interval indicating the position of the target. After another blank period of 325 ms two moving RDPs were presented peripherally. At a random time between 680–4250 ms after the onset of the RDPs, one or both of the stimuli made a direction change of 30 degrees and turned back after 130 ms. The monkey had to respond to the direction change in the target within a time window of 150–650 ms while ignoring any direction change in the distracter. The plus sign indicates the fixation point, the filled circle is the RF and the dashed circle marks the target. The circles were not presented in the experiment. B. Normalized MUA aligned to target onset. The dashed line represents the MUA recorded in the unattended condition and the solid line shows the MUA in the attended condition. Error bars show the standard error of mean (SEM).

A behaving male macaque monkey was trained to fixate to a central fixation point on the middle of the screen and keep its attention on one of two coherently moving random dot patterns (RDP) while ignoring the other. The RDPs moved in the same direction and were presented simultaneously at peripheral locations (Figure 1A).

One of the RDPs was specified as the target stimulus by a cue at the beginning of each trial. The cue was a static RDP in the same position as the upcoming target stimulus and was shown for 455 ms. The moving RDPs appeared 325 ms after the cue faded and were presented for a random period between 680–4250 ms. During this time, one or both of the stimuli could randomly make a short (130 ms) direction change of 30 degrees. The monkey was rewarded if it released the lever within a time window of 150–650 ms after the direction change of the target. If the direction change took place in the distracter, the monkey had to ignore it. Releasing the lever in these cases would lead to termination of the trial without any reward. This allowed us to ensure that the monkey switched its attention to the position of the target and ignored the position of the distracter. One of the two RDPs was placed inside the RF and the other outside. Both of them moved in the same direction in a given trial, chosen out of 8 possible directions (0 to 2π radians with steps of π/4). The preferred direction of each recorded site was determined using the direction tuning function from single unit spikes [33] that was estimated in the beginning of each session.

After the monkey reached acceptable performance in the task, multi unit activity (MUA) and LFP signals were recorded from area MT using a five-channel recording system (MiniMatrix; Thomas Recording). Spikes were sorted using the Plexon Data Acquisition System. We selected sites according to the selectivity of the isolated cells to motion direction and the position of the electrode in the cortex. Overall 112 sites were selected for analyses.

### Data Analysis

**Figure 2:**
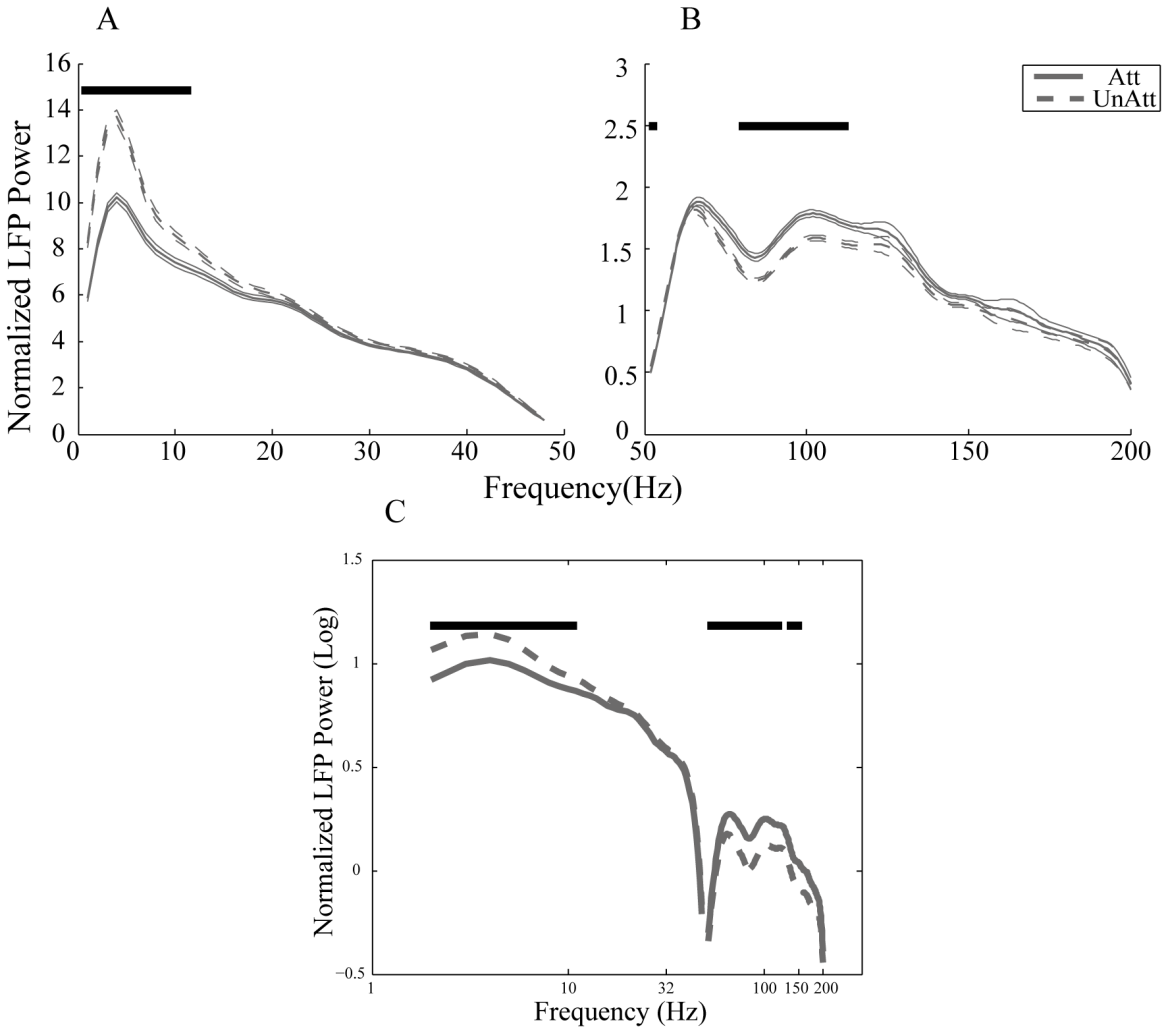
Attentional modulation of LFP power at different frequencies. LFP power at different frequencies is shown in the two attention conditions. Solid lines represent the LFP power in the attended condition and the dashed lines show the LFP power in the unattended condition. Significant differences between the LFP powers in the two conditions are indicated by stars. A. Normalized power of frequencies below 48–200 Hz. C. Logarithm of the normalized power for 1–200 Hz excluding 48–52 Hz. Error bars show SEM.

All analyses were carried out using MATLAB (Mathworks, Natick, MA). To generate the spike density function for the two attention conditions (Figure 1B) we convolved a Gaussian function (sd = 15) with the MUA recorded across trials of each condition per site and normalized them to the maximum across the two conditions for each site. We aligned the phases of LFP signals in order to correct the phase lags enforced by the recording hardware [34]. The mean of each LFP signal was subtracted from it in order to cancel the DC factor. The 50 Hz noise, 76 Hz noise due to the monitor refresh rate and its periodical (152 Hz) were band-pass filtered and removed using EEGLAB toolbox [35]. We calculated the power spectral density (PSD) of each signal by taking the absolute value of the Fourier transform applied to the signal. The PSDs were averaged across the trials of each site. For presentation purposes (Figure 2), the PSD calculated for each site was smoothed by convolution with a Gaussian kernel with sd = 2 for <50 Hz frequencies and sd = 5 for >50 frequencies. The reason we used a larger kernel for >50 Hz frequencies was that they showed a noisier trend than lower frequencies. Each trial-averaged PSD was normalized by the average PSD across the attention conditions for each site. We corrected for multiple comparisons in Figure 2 by using Bonferroni correction and assumed the p-value threshold of 0.01 to decide about statistical significance.

To classify the PSDs into the two classes of attention, we used a support vector machine (SVM) algorithm. This algorithm estimates the hyper-plane that can best separate the data points of the two classes from each other [36]. To be more explicit, SVM finds the terms w and b in this equation: y_i_(<w,x_i_>+b)≥1; in a way that ‖‖w‖‖ (norm of w) is minimized and the equation holds true for all data points (x_i_,y_i_) where x_i_ is the data point number i and y_i_ is the class number for the corresponding data point that changes between −1 and 1. The symbol “< >” means the dot product function and *w* represents the weights of the dimensions for each data point. We used the default SVM algorithm developed in MATLAB with a linear kernel. We first averaged the PSDs across trials for each recording site. In order to generate a distribution of classification performances for a given number of trial-averaged PSDs, we randomly divided the data set of all trial-averaged PSDs into train and test subsets 500 times and each time we trained the classifier using the train data and calculated the performance using the test data. In order to find out if a given distribution of performance is significantly above 50% (chance level), we checked if the mean of the distribution was more than 2*sd apart from 50%, which would be equivalent to the confidence interval of 95%.

To calculate the ROC curves, we used the dot product of *w* and the data point vectors for different frequency bands. We randomly selected 80% of the data for computing the *w* vector and applied the weight vector to the remaining test data point. This procedure was repeated 50 times to calculate the variability of the ROC curves. The coefficient of variation for each frequency point was calculated by dividing the standard deviation of the power of that frequency by its mean power across sites.

## Results

We trained a monkey to detect a small change in the motion direction of one of two moving RDPs (target) and to ignore the other one (distracter). The monkey responded correctly to the direction change of the target and ignored the distracter in 86% of trials that were accomplished without any fixation break. The monkey had to attend inside the RF in half of the trials and outside the RF in the other half. We focused our analyses on the period between the onset and the direction change of the target RDP. Figure 1B shows the multi unit activity (MUA) after the target onset for the two conditions in which the monkey attended inside RF (attended) or outside RF (unattended). The solid and dashed curves represent the attended and unattended conditions respectively. It is obvious that the two curves become separated soon around 350 ms after the target is presented and stay apart as time passes (p≪0.01 for all time points between 350–1400 ms; paired t-test). This shows that the monkey has attended to the target and ignored the distracter [3]. Also we observe that the distance between the two curves slightly increases as time passes. This might be due to the fact that before any direction change occurs, the probability of direction change occurrence increases as time passes; therefore the monkey's expectation might become higher approaching the end of the trial. However the effect of attention remains across time.

Figure 2A&B show the power of LFP signals at different frequencies for the attended and unattended conditions. We showed the results in two separate bands, one within 1–48 Hz and one within 52–200 Hz due to the 50 Hz noise (Note that the dimensions differ between the two figures). As it is shown in Figure 2C, the 1/f relationship across the power of different frequencies can be seen clearly for both attention conditions, which is consistent with previous reports [6]. The curves corresponding to the two attention conditions diverge for frequencies less than 12 Hz and between 80–112 Hz (Figure 2). Attending inside the RF is associated with a decrease in LFP power within frequencies less than 12 Hz, while it is associated with the increase of LFP power within the high frequencies of 80–112 Hz which correspond to the gamma band. These two effects are consistent with previous reports suggesting the decrease of low frequency synchrony and increase of high frequency synchrony in the visual cortex with attention [8]–[10]. See also [13] for different results in V1 within the gamma band.

**Figure 3:**
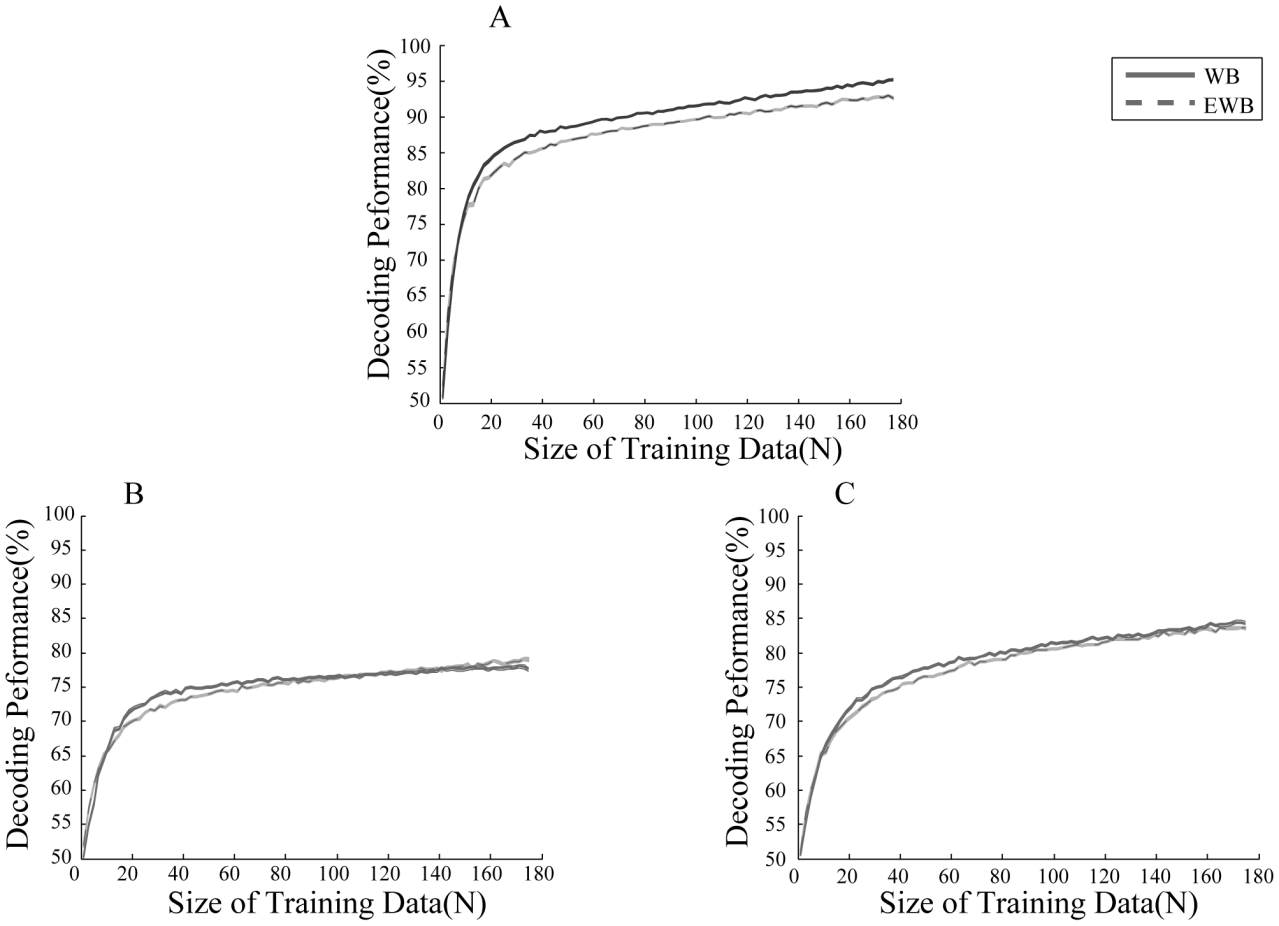
Performance of decoding attention condition using LFP power at different frequencies. A. Decoding performance for trials in which any of 8 possible directions (0 to 2π radians with steps of π/4) were presented. B. Decoding performance for trials in which the preferred direction was shown as the target. C. Performance for trials with the anti-preferred direction as the target. Performances are shown given different sizes of training data. The solid lines indicate the performance using LFP powers between 1–200 Hz (marked as WB) and the dashed lines show the performance using LFP powers between 1–500 Hz (marked as EWB). Error bars show SEM.

To investigate whether the power of LFP at different frequencies could help in decoding attentional conditions, we calculated the PSD of LFP signals between 400–1400 ms after target onset within the frequency range of 1–500 Hz for each trial per recoding site. The PSDs were then averaged across trials for each condition separately. This gave us a set of 224 PSDs, half of them corresponding to the attended condition and the rest corresponding to the unattended condition. We randomly selected a subset of these signals consisting of an equal number of signals for the two conditions and used them to train a linear SVM to classify the conditions of attention. We tested the trained algorithm with the rest of the PSDs and calculated the classification performance by dividing the number of correct classifications by size of the test data. Figure 3 shows the classification performance for different numbers of training data from 2 data points up to 178 data points (equivalent to 80% of the data). We also calculated the classification performance for the limited band of 1–200 Hz since it is largely assumed that LFP information is mostly limited to frequencies less than 200 Hz. Figure 3A shows the decoding performance for different number of training data for trials in which the target moved in any of the 8 possible directions. The performances were calculated using the wide band (WB) (1–200 Hz) and the extended wide band (EWB) (1–500 Hz) LFP separately. Similarly Figure 3B&C show the decoding performance for trials in which the target moved in the preferred or anti-preferred direction respectively. Figure 3A shows that WB outperforms the EWB across a variety of sizes for training data. The maximum performance across the two cases differs significantly (p≪0.001 t-test). This is consistent with the assumption that the WB band contains the major part of the LFP information. However since the EWB band contains all the information of the WB band, we would expect that the EWB curve would outperform the WB curve given more training data. For the trials with targets moving in the preferred or anti-preferred direction (Figure 3B&C) the maximum performance decreases compared to the case of all directions, which is due to the lack of trials (p≪0.001 for both conditions; t-test). For trials with the target moving in the preferred direction however the EWB maximum performance is higher than the WB maximum performance (p<0.001) (Figure 3B). This suggests that there is some extra information in the 200–500 Hz band that can help decoding when the target is preferred. Similar to the trials with all directions, we found that for trials with anti-preferred targets, the maximum performance in the WB band was only 0.8% higher than the EWB band (p<0.01) (Figure 3C).

**Figure 4:**
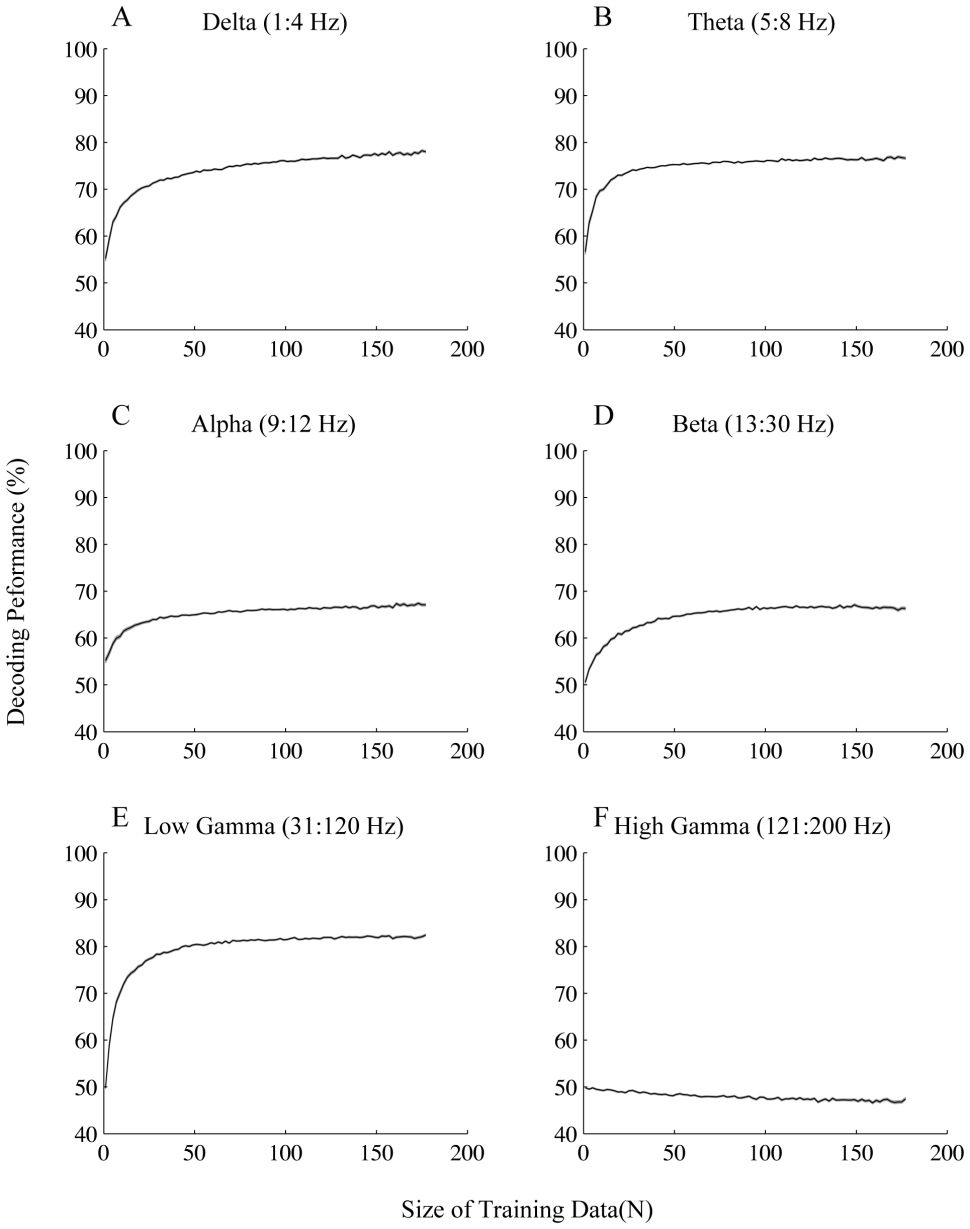
Performance of decoding for trials with the target moving in any of the 8 equally separated directions. Each plot shows the decoding performance for the frequency band written above it given different sizes of training data. Error bars represent SEM.

**Figure 5:**
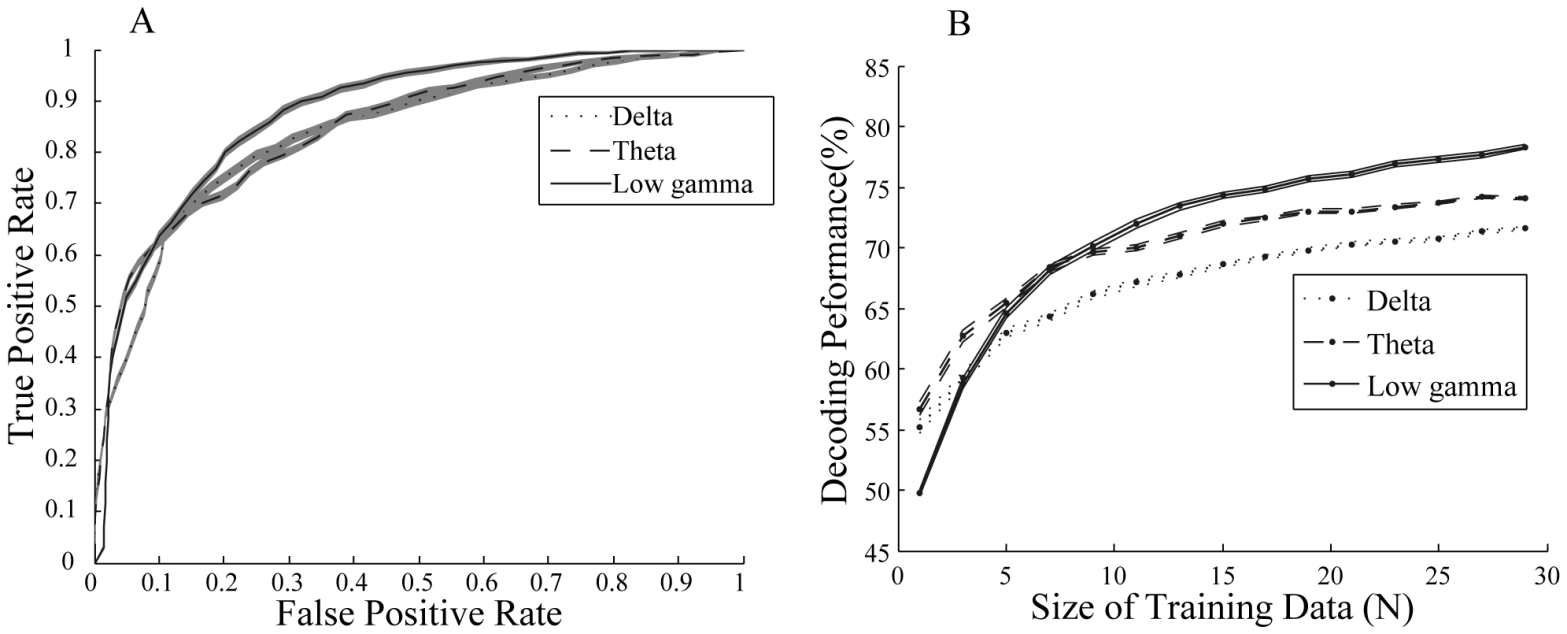
Comparison of decoding across delta, theta and low gamma bands. A. ROC curves of the low frequency bands (delta & theta) vs. low gamma band using the features extracted by the SVM algorithm. B. The learning curve of the three bands limited to the size of training data less than 30. Delta, theta and low gamma bands are represented using dotted, dashed and solid lines respectively. Error bars show SEM.

We then asked which frequency band contributed most to the training of the learning algorithm by applying the classification process explained above on different frequency bands. The traditional bands were used for this purpose: delta (1–4 Hz), theta (5–8 Hz), alpha (9–12 Hz), beta (13–30 Hz) and gamma (31–200 Hz). We further divided the gamma band into two separate bands for a more detailed study: low gamma (31–120 Hz) and high gamma (121–200 Hz). Figure 4 shows the performance results for the frequency bands separately, for all motion directions. All frequency bands except high gamma reach a performance significantly above chance level (50%) with quite a small number of training data (a maximum of 18 data points) (See Materials and Methods for details). Low gamma reached the highest performance compared to the other bands (maximum of 82%±5 (SD)). This suggests that gamma band activity has the largest contribution to the decoding of attention condition. This is consistent with the attentional modulation of LFP power observed within 80–112 Hz (Figure 2) and also previous findings emphasizing the role of gamma oscillations in attentional processing [37]. The next highest performances correspond to delta and theta bands with 78%±5 (SD) and 77%±5 (SD) respectively that are both significantly smaller than the highest performance for low gamma (p≪0.001 t-test). In order to justify the difference in performance across the low and high frequency bands, we plotted the ROC curves based on the features that the SVM algorithm extracted for each band (Figure 5A). We calculated these features by applying the SVM weight assigned to each frequency (Figure S1) (See Materials and Methods for details). It is obvious that the area under the ROC curve for the low gamma band is larger than delta and theta bands (p<0.01). This is consistent with the finding that SVM gives a higher decoding performance for low gamma band compared to delta and theta bands. Next we looked at the speed at which the low frequency and high frequency bands converge to their highest performance. Figure 5B shows the learning curve for delta, theta and low gamma bands limited to small sets of training data (with less than 30 data points). Delta and theta bands have higher performances for training sets of size below 3 and 5 (respectively) compared to low gamma band (p≪0.001 corrected for multiple comparison). Noticeably low gamma does not reach a significantly higher performance than theta with any data size smaller than 11 (Figure 5B). This suggests that delta and theta bands can reach higher performances compared to the low gamma band with quite a small amount of training data although low gamma finally reaches a performance greater than that of both delta and theta. This effect could be a consequence of differences in signal variability across the different bands. We therefore calculated the coefficient of variation across the recording sites for each of the bands (See Materials and Methods for the details). The coefficient of variation in low gamma band was significantly higher than that of both delta and theta bands (p<0.01; Wilcoxon rank sum test) (Table 1). In order to control for any potential effect of the cue, we did the same analyses within the period 700–1700 ms after the target onset which is far enough from the offset time of the cue. Similar results were observed in this period, which rules out any potential effect of cue on the results (Figure S2).

**Table 1:** Peak value of decoding performance and coefficient of variation for different LFP bands in trials with any of the 8 motion directions.

Frequency Band	Performance (%) (±SD)	Coefficient of Variation (±SD)
Delta (1–4 Hz)	78 (±5)	6.8 (±9)
Theta (5–8 Hz)	77 (±5)	2.7 (±1)
Alpha (9–12 Hz)	67 (±6)	2.7 (±0.5)
Beta (13–30 Hz)	67 (±5)	11.2 (±10)
Low Gamma (31–120 Hz)	82 (±5)	16.7 (±31)
High Gamma (121–200 Hz)	50 (±2)	335.4 (±2293)

**Figure 6:**
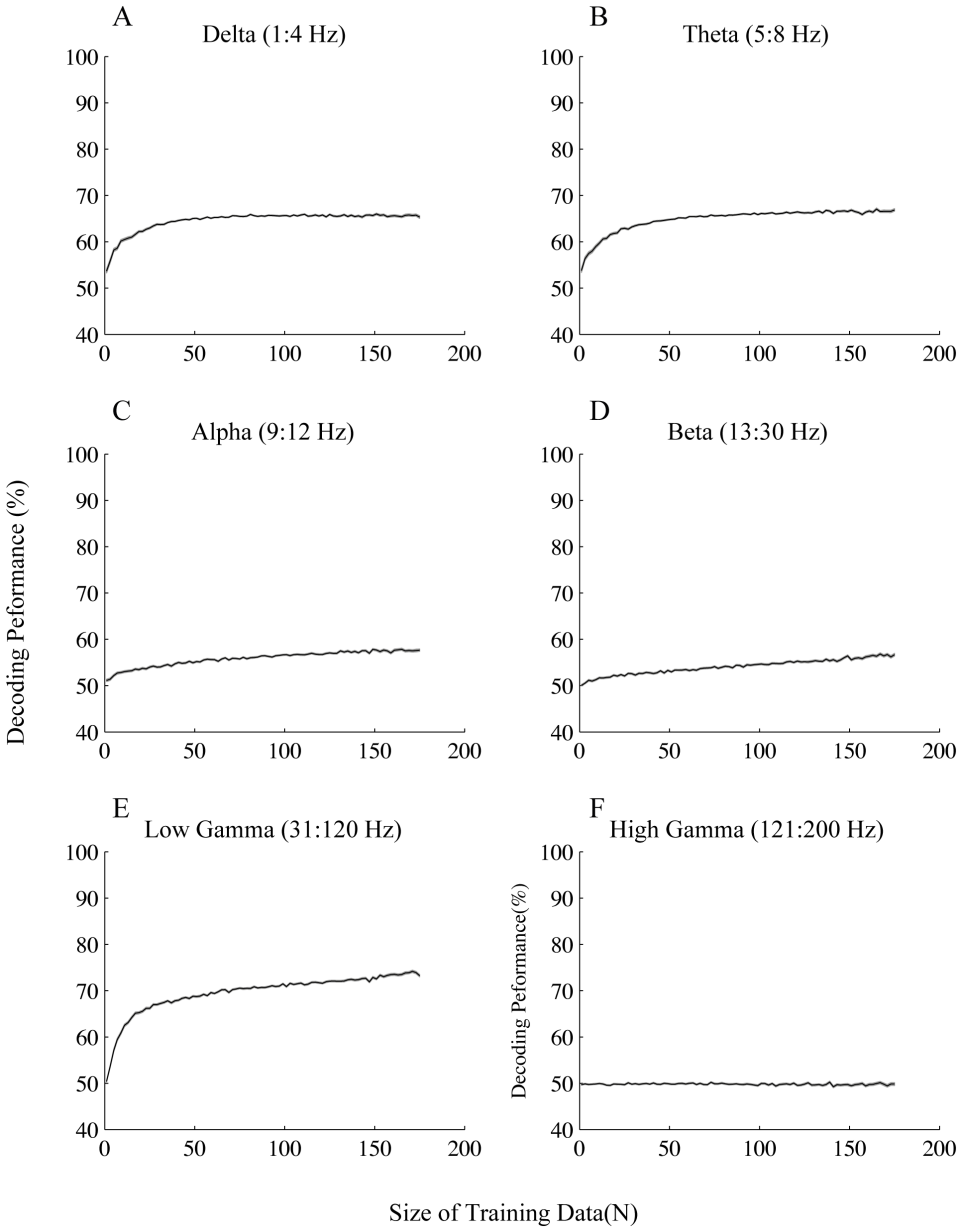
Performance of decoding for trials with the target moving in the preferred direction. Each plot shows the decoding performance for the frequency band written above it given different sizes of training data. Error bars represent SEM.

**Figure 7:**
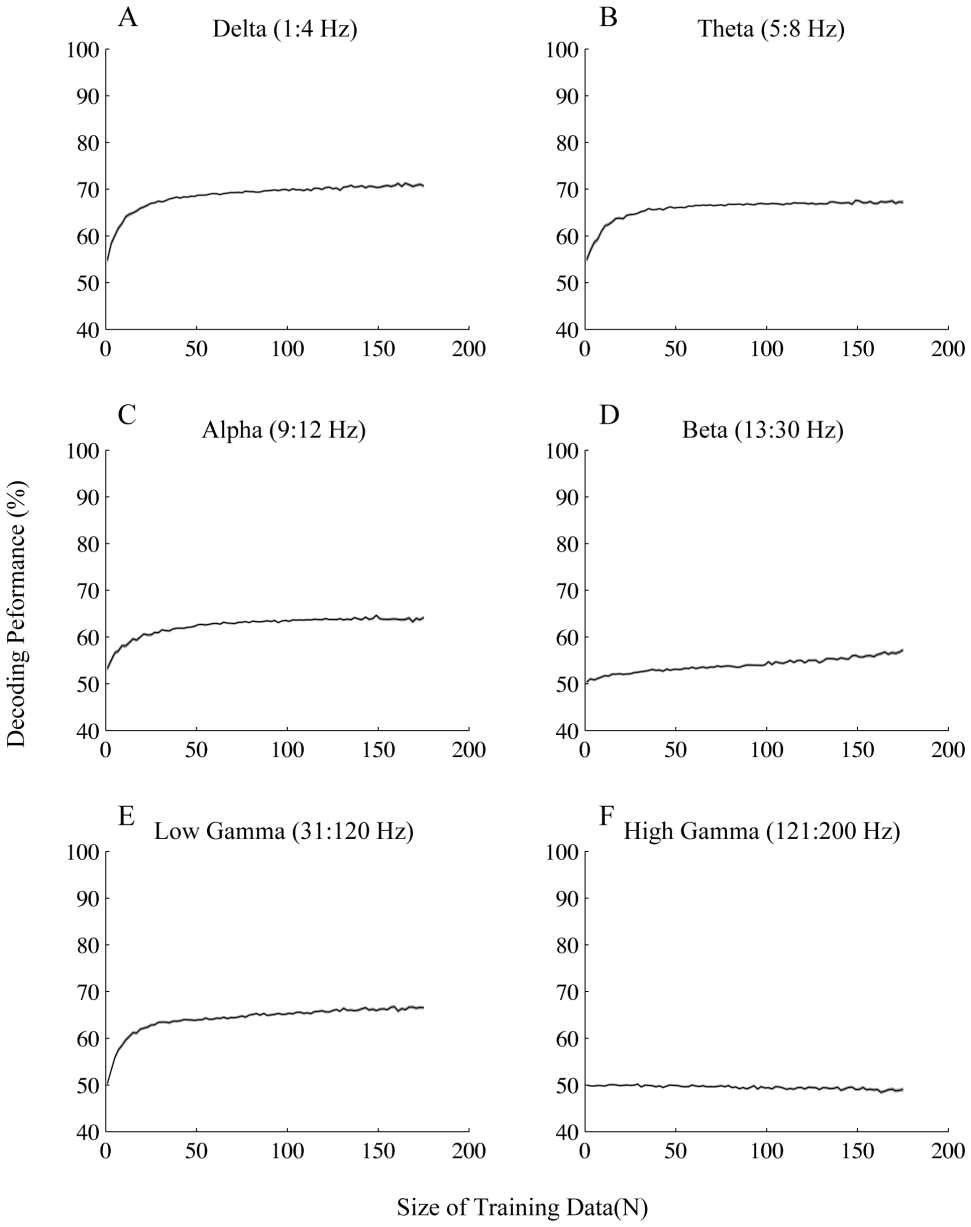
Decoding performance for trials with the target moving in the anti-preferred direction. Each plot shows the decoding performance for the frequency band written above it given different sizes of training data. Error bars represent SEM.

Next we asked if the preferred vs. anti-preferred directions would differ in terms of decoding performance in the different frequency bands. Therefore we carried out the same analyses described above on trials in which the stimuli moved in the preferred and anti-preferred direction of the recorded sites (Figure 6 & Figure 7 respectively; see Materials and Methods for the details of choosing preferred vs. anti-preferred directions). Consistent with the case of all directions (Figure 4), for the preferred direction delta, theta and low gamma bands reached a performance significantly above chance level (50%) with less than 30 data points. Low gamma band again reached the highest performance compared to the other bands (maximum of 74%±6 (SD)). Similar to what we observed for all directions, we found that both delta and theta bands reached significantly higher performances compared to low gamma with less than 5 training data (p≪0.001 Wilcoxon rank sum test). This effect was also reflected in the differences between the coefficients of variation calculated for each band (Table 2), i.e. coefficient of variation across recording sites was significantly smaller for low frequency bands (delta and theta) compared to low gamma (p<0.01 Wilcoxon rank sum test).

**Table 2:** Peak value of decoding performance and coefficient of variation for different LFP bands in trials with preferred vs. anti-preferred targets.

	Performance (%) (±SD)			Coefficient of Variation (±SD)	
Frequency Band	Preferred Target	Anti-Preferred Target	P-value (t-test)	Preferred Target	Anti-Preferred Target
Delta (1–4 Hz)	66 (±5)	71 (±5)	≪0.001	4.6 (±3)	8.5 (±11)
Theta (5–8 Hz)	67 (±5)	68 (±4)	>0.05	8.0 (±9)	2.9 (±1)
Alpha (9–12 Hz)	58 (±6)	65 (±5)	≪0.001	7.9 (±3)	3.7 (±1)
Beta (13–30 Hz)	57 (±6)	57 (±7)	>0.1	22 (±20)	25 (±37)
Low Gamma (31–120 Hz)	74 (±6)	67 (±5)	≪0.001	44 (±147)	54 (±260)
High Gamma (121–200 Hz)	50 (±5)	50 (±3)	>0.1	172 (±1166)	80.6 (±138)

For the anti-preferred case, it is specifically remarkable that the decoding performance in the frequencies of low gamma range significantly decreased by 7% compared to the case of preferred targets: According to Table 2, the peak performance for low gamma range was 74%±6 (SD) for trials with preferred targets, while it decreased to 67%±5 (SD) in the same band for trials with anti-preferred targets (p≪0.001 t-test). Conversely delta and theta bands increased or did not change their maximum decoding performance when the direction switched to anti-preferred; Delta band had a 5% increase in its maximum performance (p<0.001 t-test), but theta band did not have any significant change in its maximum performance (p>0.05 t-test). Similar to delta band, we found that alpha had an increase of 7% in its peak performance compared to the case of preferred targets (p≪0.001 t-test) (Table 2).

## Discussion

We found that the focus of attention can be decoded using the power of the LFP in a wide range of frequencies. This is consistent with previous findings regarding the influence of spatial attention on the power of LFP at different frequencies in the visual cortex [9], [10], [13]. This is also in agreement with studies that have shown an increase of decoding performance in the visual cortex by attention [29], [38]. It is further compatible with the high performance for decoding attention using ECoG signals reported by Rotermund et al. [32], especially the high performance of decoding at the high frequencies. However Rotermund et al. [32] did not report any significant decoding power for low frequency bands (<30 Hz), while we reported here that low frequency bands (<30 Hz) can reach the performance of 78% (Table 1). This suggests that even LFP signals at frequencies as low as delta band contain information about attention which may be due to the locality of LFP signals relative to ECoG signals [5], [39].

The highest decoding performance across the traditional frequency bands was observed in low gamma. Previous reports have shown that gamma power is linked to spiking activity in the visual cortex [40], [41] which is thought to be associated with communication between cortical areas [37]. Therefore the high performance of low gamma power might be related to the information coded in spike rate which was also observed earlier in the inferior temporal cortex [29]. We also found that the performance of decoding attention using low gamma is significantly higher when the target is preferred relative to anti-preferred. This could be a result of the reduced effect of spontaneous noise on the neural response to preferred stimuli, since neurons fire at a higher rate in response to preferred target. On the other hand due to the multiplicative effect of attention on neural firing [42], the discrimination between the responses in the two conditions becomes easier when the stimulus is preferred. Conversely the decoding performance at low frequencies (delta and alpha bands) was significantly higher for the anti-preferred direction compared to the preferred direction (Table 2). This suggests that attention has more influence on the power of low frequency LFPs when evoked by anti-preferred rather than preferred stimuli.

Despite the weaker decoding performance for the low frequency bands relative to low gamma band, we found that the power of low frequency bands (<9 Hz) could help the classifier reach a higher decoding performance with a low number of training data compared to low gamma band. This suggests that BCIs could use the low frequency power to gain some coarse information about the coding strategy of the brain given just a few sample trials and further achieve a more accurate learning of the code within the rest of trials.

Using the power of wide band LFPs (1–500 Hz), our classifier reached the performance of 79% when considering the preferred direction (Figure 3B). However when we used only the limited wide band of 1–200 Hz we achieved lower performances with very low number of data points (<7). Also we observed that the values of the 1–500 Hz curve went higher than the 1–200 Hz curve especially at the 2 highest numbers of training data points (p<0.05 t-test) (Figure 3B). This would suggest that there is some extra information about attentional state in the power of frequencies higher than 200 Hz. This information might be related to spikes as proposed before [43].

## Conclusions

In this study we showed that attention can be decoded using the power of LFP signals recorded from area MT of macaque monkey. Low gamma band has the strongest contribution to the decoding performance especially when the preferred target of the recorded site is presented. It was found that the power of low frequencies (<9 Hz) can be used for decoding attention with very few training data. We finally suggest that the decoding performance may be improved if spiking data were to be incorporated into the LFP power.

## Supporting Information

Figure S1 **SVM weights given to different frequencies of each band.** These weights were calculated by training the SVM with 80% of the dataset. The variation of each weight was calculated by randomly selecting the training data 50 times.
(TIF)

Figure S2 **Decoding performances for different numbers of training data for all the 8 possible directions within the period 700–1700 ms after the target onset.** Each plot presents the performance for the frequency bands written above it. Error bars represent SEM.
(TIF)
