# Supplementary figures and images for: Decoding of Visual Attention from LFP Signals of Macaque MT
Source: PLoS One. 2014 Jun 30;9(6):e100381. doi: 10.1371/journal.pone.0100381 (PMC4076262; doi:10.1371/journal.pone.0100381)

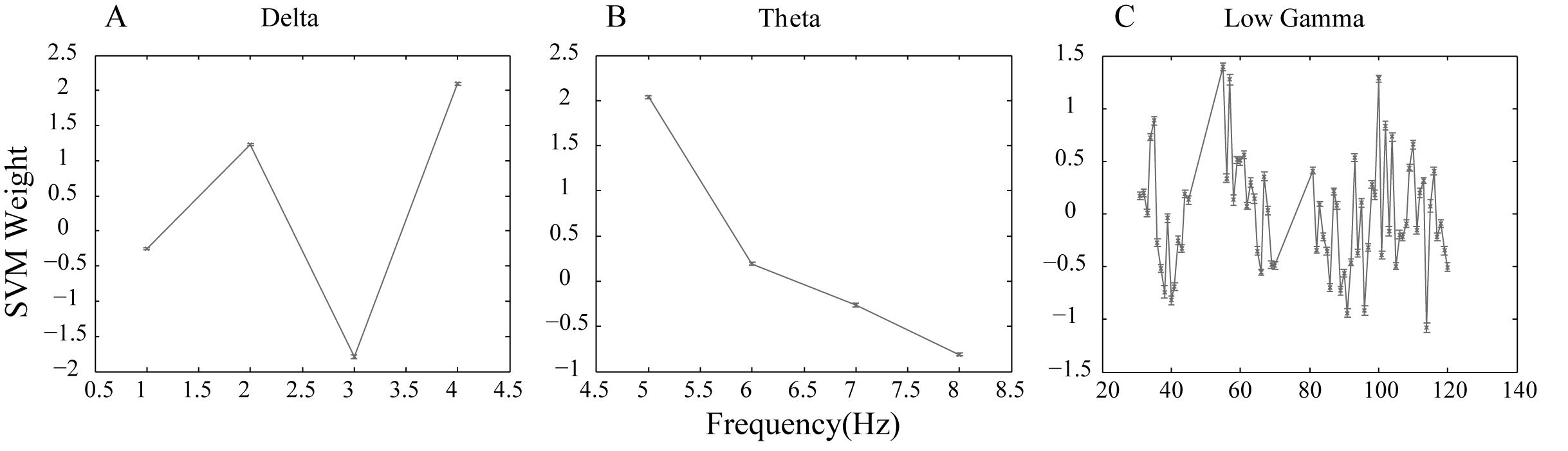

Supplement: Figure S1 — SVM weights given to different frequencies of each band. These weights were calculated by training the SVM with 80% of the dataset. The variation of each weight was calculated by randomly selecting the training data 50 times. (TIF) [file pone.0100381.s001.tif]

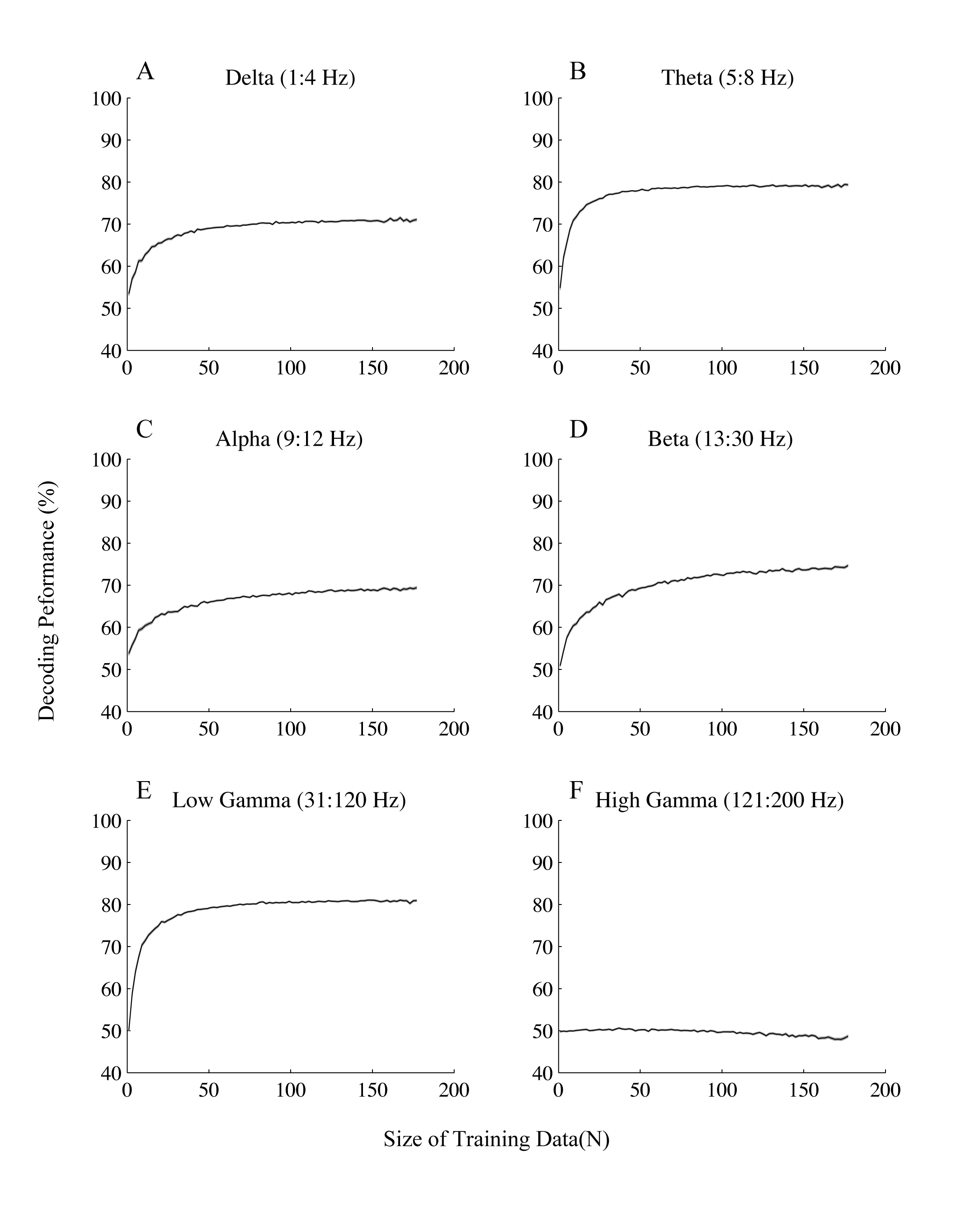

Supplement: Figure S2 — Decoding performances for different numbers of training data for all the 8 possible directions within the period 700–1700 ms after the target onset. Each plot presents the performance for the frequency bands written above it. Error bars represent SEM. (TIF) [file pone.0100381.s002.tif]
